# The first genome sequences of human bocaviruses from Vietnam

**DOI:** 10.12688/wellcomeopenres.10042.2

**Published:** 2017-01-09

**Authors:** Tran Tan Thanh, Hoang Minh Tu Van, Nguyen Thi Thu Hong, Le Nguyen Truc Nhu, Nguyen To Anh, Ha Manh Tuan, Ho Van Hien, Nguyen Manh Tuong, Trinh Trung Kien, Truong Huu Khanh, Le Nguyen Thanh Nhan, Nguyen Thanh Hung, Nguyen Van Vinh Chau, Guy Thwaites, H. Rogier van Doorn, Le Van Tan

**Affiliations:** 1 Oxford University Clinical Research Unit in partnership with the Hospital for Tropical Diseases, Ho Chi Minh, 700000, Vietnam; 2 Children’s Hospital 2, Ho Chi Minh, 700000, Vietnam; 3 Children’s Hospital 1, Ho Chi Minh, 700000, Vietnam; 4 Hospital for Tropical Diseases, Ho Chi Minh, 700000, Vietnam; 5 Centre for Tropical Medicine, Nuffield Department of Medicine, University of Oxford, Oxford, OX3 7DQ, UK

**Keywords:** human bocavirus, HBoV, HFMD, Vietnam

## Abstract

As part of an ongoing effort to generate complete genome sequences of hand, foot and mouth disease-causing enteroviruses directly from clinical specimens, two complete coding sequences and two partial genomic sequences of human bocavirus 1 (n=3) and 2 (n=1) were co-amplified and sequenced, representing the first genome sequences of human bocaviruses from Vietnam. The sequences may aid future study aiming at understanding the evolution of the virus.

## Introduction

Human bocaviruses (HBoV) are non-enveloped, single stranded DNA viruses of the family *Parvoviridae,* subfamily *Parvovirininae* and genus *Bocaparvovirus.* The virus genome is ~5.3 Kb in length. HBoV-1 was first discovered in 2005 ^1^. Since then three additional HBoV species, namely HBoV-2, HBoV-3 and HBoV-4, have been discovered ^2, 4^. While the clinical significance of HBoV remains unknown, worldwide their prevalence in respiratory/gastrointestinal tracts varies between 0–26% ^5, 6^. In Vietnam, the reported prevalence of HBoV was 2–17% ^7– 10^. Currently, there is relatively limited sequence information, especially at genome-wide level, of HBoV from Vietnam, although such knowledge may be essential for the development of sensitive, specific diagnostic PCR for the local viral strains, and may aid future investigation documenting the circulation and spread of the viruses at global scale. 

Herein we report the recovery of two complete coding sequences (CDS) and two partial genomic sequences of HBoV from swabs of Vietnamese children enrolled in our ongoing hand, foot and mouth disease (HFMD) research program in Ho Chi Minh City. The research program aims to look at various disease aspects, including pathogen evolution and its potential implication for vaccine development and implementation.

## Methods and results

Whole-genome sequencing of the dominant pathogens (including coxsackievirus A6 (CV-A6), CV-A10 and CV-A16) were performed on 296 RT-PCR positive swabs using an in-house MiSeq-based approach ^11^. In brief, 110 µl of selected swabs were centrifuged at 13,500 rpm for 10 minutes to remove host cells or large cellular components. After DNAse treatment, viral nucleic acid (NA) was then isolated from 100 µl of supernatant using QIAamp viral RNA kit (QIAgen GmbH, Hilden, Germany), and recovered in 50 µl of elution buffer (provided with the kit). Ten microliter of the isolated NA was subjected to cDNA synthesis using Super Script III kit (Invitrogen, Carlsbad, CA, USA) and FR26RV-Endoh primer (primer sequences can be found elsewhere ^11^). The cDNA was then converted to double-stranded DNA using exo-Klenow (Invitrogen), and subsequently pre-amplified using Platinum PCR supermix (Invitrogen) and FR20RV primer ^11^. PCR product was then purified and subjected to library preparation using Nextera XT DNA sample preparation kit (Illumina, San Diego, CA, USA) and was finally sequenced using MiSeq reagent kits (Illumina) in an Illumina MiSeq platform (Illumina) ^11^.

After reference-based mapping ^11^ to generate the complete genome sequences of the targeted enteroviruses using Geneious software v 8.1.5 (Biomatters, Ltd, Auckland, New Zealand), the remaining reads were then subjected to publicly available metagenomic pipelines; Taxonomer ^12^ and Sequence-based Ultra-Rapid Pathogen Identification (SURPI) ^13^ to explore the contents of non-enteroviral sequences in the tested swabs. Evidence of bocavirus sequences were found in four swabs (including 3 throat- and 1 rectal swabs). A reference-based mapping approach using Geneious software (Biomatters) ^11^ was then employed to recover the HBoV genomes from the corresponding dataset. Subsequently, 2 CDS (1 from a throat swab with 4925 bp in length and the other from a rectal swab with 4898 bp; i.e. over 90% of genome coverage) were successfully assembled with a mean coverage of 1,922 and 3,745, respectively. In the other two datasets from the remaining 2 swabs only partial genomic sequences of HBoV, each with 2870 bp in length and a mean coverage of 15.4 and 448.7, were recovered.

**Figure 1.**
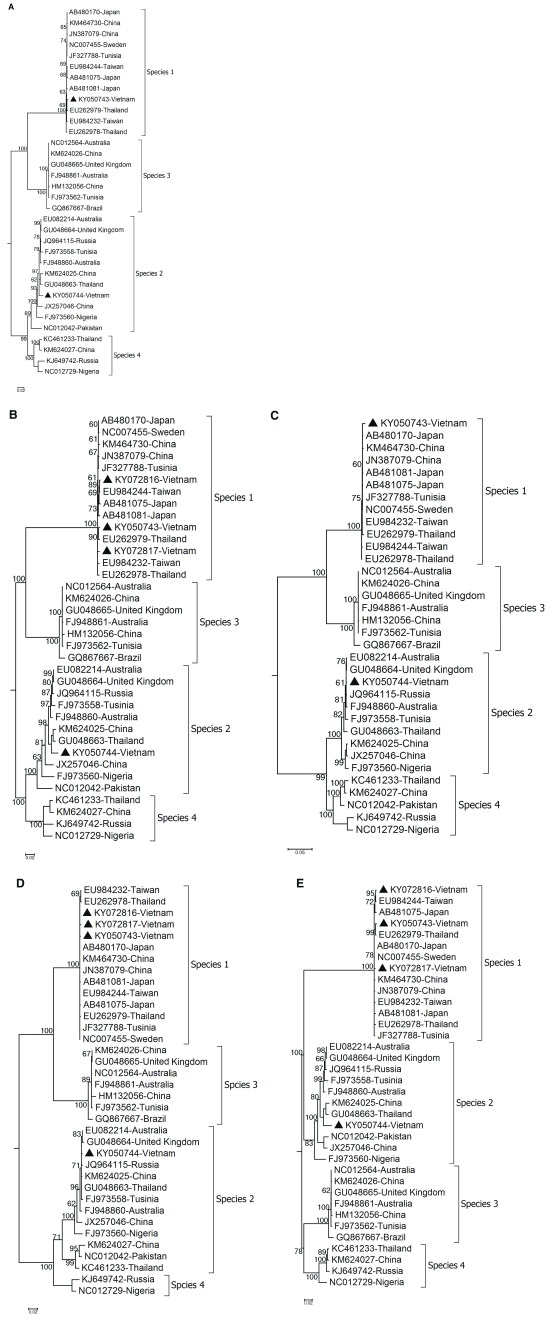
Phylogenetic trees showing the relationship between the Vietnamese bocaviruses and representative worldwide circulating strains. **A**) Neighbor-joining phylogeny of CDS; **B**) Neighbor-joining phylogeny of partial genomic sequences spanning the region from nucleotide 1897 to 4856 of the HBoV genomes; **C**) Neighbor-joining phylogeny of ORF1 encoding NS1 protein; **D**) Neighbor-joining phylogeny of ORF2 encoding NP1 protein; **E**) Neighbor-joining phylogeny of ORF3 encoding VP1 and VP2 proteins. Trees were reconstructed using Neighbor-joining method available in Geneious with Tamura-Nei nucleotide substitution model, and support for individual nodes was assessed using a bootstrap procedure (1000 replicates). Bootstrap values greater than 60% are shown on the branch nodes. The Vietnamese strains from this study are indicated by solid triangles. The scale bars indicate the number of nucleotide substitution.

Subsequent sequence alignment and phylogenetic analysis using MUSCLE ^14^ and Neighbor-joining available in Geneious (Biomatters), respectively ( Figure 1) revealed that all 3 Vietnamese HBoV recovered from the throat swabs belonged to HBoV-1 and had >98% of sequence similarity at nucleotide level with other HBoV-1. The other belonged to HBoV-2 and had a close relatedness with a Thai strain CU54TH (GU048663) with a sequence similarity of 97.3% ( Figure 1). Similar results were obtained when the analyses were done for 3 individual open reading frames (ORF1, ORF2, and ORF3) of the virus genome ( Figure 1).

All the four HFMD patients (including 3 CV-A6 and 1 CV-A12, Table 1) in whom HBoV was detected had mild HFMD, and were enrolled in November 2013 – March 2014. Three had vomiting, and two presented with runny nose and cough ( Table 1).

**Table 1.** Demographic, clinical information, and details of MiSeq sequencing results.

Patient	Gender	Age (year)	Admission date	HFMD grade	Respiratory/gastrointestinal signs/ symptoms				MiSeq sequencing results							
									Sample	Enterovirus			Bocavirus			
					Runny nose	Cough	Vomiting	Diahrrea		Serotype	Full genome	Mean coverage	Species	Complete CDS	Mean Coverage	Genbank accession
1	Male	0.9	11/3/2014	2a	Yes	Yes	Yes	No	Throat swab	CV-A6	Yes	6,024	1	Yes	1,922	KY050743
2	Male	0.4	5/11/2013	2a	No	No	No	No	Rectal swab	CV-A6	Yes	12,303	2	Yes	3,745	KY050744
3	Male	1.4	14/11/2013	1	No	No	Yes	No	Throat swab	CV-A12	Yes	2,841	1	No	15.4	KY072816
4	Female	0.8	4/12/2013	1	Yes	Yes	Yes	No	Throat swab	CV-A6	Yes	2,493	1	No	448.7	KY072817

## Discussion

Herein we reported for the first time 2 complete CDS alongside two other partial genomics sequences of HBoV from Vietnam. Phylogenetically, the four HBoVs from Vietnam were closely related to other HBoV strains sampled from various countries worldwide, reflecting a wide distribution of these HBoV lineages at global scales.

All three HBoV detected in throat swabs belong to species 1, while the remaining virus detected in rectal swab was HBoV-2. This is in line with previous reports regarding the frequent detection of HBoV-1 and HBoV-2 in throat- and rectal swab, respectively ^5, 6, 15– 18^, albeit our sample size was small. Likewise, all the four HFMD patients in whom HBoVs were found were enrolled into our HFMD study during the seasonal peak of HBoV in southern Vietnam ^8^.

Although the pathogenic potential of HBoV infections remains unknown, clinical signs/symptoms such as vomiting, runny nose and cough were also commonly recorded among HFMD patients in previous reports ^19– 21^. HBoV has commonly been co-detected with other pathogens in respiratory and gastrointestinal tracts ^5, 10, 16– 18^. It was also previously detected in fecal samples of HFMD patients from Thailand ^22^. Clearly, further research is needed to ascribe the contribution of coinfections to clinical manifestation and pathogenesis of HFMD. Of note, previous reports showed that there might be an association between coinfections with other viral pathogens such as norovirus and rotavirus and clinical severity of HFMD patients ^23^.

In conclusion, to the best of our knowledge, we are the first to report the complete CDS of HBoVs from Vietnam. The contribution of HBoV to clinical manifestation of HFMD requires further research.

## Data availability

**Nucleotide sequence accession numbers**: the HBoV sequences have been submitted to Genbank ( https://www.ncbi.nlm.nih.gov/genbank/) under accession numbers KY050743, KY050744, KY072816 and KY072817.

## Consent

The clinical samples used in this study were derived from an on-going HFMD study in three referral hospitals in Ho Chi Minh city, Vietnam. The study was reviewed and approved by the local Institutional Review Boards and the Oxford Tropical Research Ethics Committee (OxTREC), University of Oxford, Oxford, United Kingdom. Written informed consent was obtained from parent or legal guardian of each participant.
