# Supplementary material for: The first genome sequences of human bocaviruses from Vietnam
Source: Wellcome Open Res. 2017 Jan 9;1:16. Originally published 2016 Nov 16. [Version 2] doi: 10.12688/wellcomeopenres.10042.2 (PMC5234701; doi:10.12688/wellcomeopenres.10042.2)
Supplement: Peer review history

# Peer review: Referee response for version 2

**DOI:** 10.21956/wellcomeopenres.11425.r19091

**Published:** 2017-01-13

**Authors:** Verena Schildgen, Oliver Schildgen

**Affiliations:** 1 Department of Pathology, gGmbH Clinic of Cologne, Hospital of University of Witten/Herdecke, Cologne, Germany

All previous comments have been thoroughly addressed and the manuscript has significantly improved.

We have read this submission. We believe that we have an appropriate level of expertise to confirm that it is of an acceptable scientific standard.

# Peer review: Referee response for version 2

**DOI:** 10.21956/wellcomeopenres.11425.r19090

**Published:** 2017-01-12

**Authors:** Bas B. Oude Munnink

**Affiliations:** 1 Viroscience, Erasmus MC, Rotterdam, Netherlands

The authors have carefully addressed all comments raised in the previous review.

I have read this submission. I believe that I have an appropriate level of expertise to confirm that it is of an acceptable scientific standard.

# Peer review: Referee response for version 2

**DOI:** 10.21956/wellcomeopenres.11425.r19089

**Published:** 2017-01-11

**Authors:** Xiu-ling Ji

**Affiliations:** 1 Faculty of Life Science and Technology, Kunming University of Science and Technology, Kunming, China

I suggest this manuscript can be indexed.

I have read this submission. I believe that I have an appropriate level of expertise to confirm that it is of an acceptable scientific standard.

# Peer review: Referee response for version 1

**DOI:** 10.21956/wellcomeopenres.10819.r18202

**Published:** 2016-12-05

**Authors:** Xiu-ling Ji

**Affiliations:** 1 Faculty of Life Science and Technology, Kunming University of Science and Technology, Kunming, China

Human bocavirus (HBoV) was first identified in 2005, and regarded as a causative pathogen of respiratory tract diseases. The paper reported the recovery of two complete coding sequences and two partial genomic sequences of HBoV from swabs of Vietnamese children enrolled in HFMD research program in Ho Chi Minh City. The experiments were well designed and performed. In addition, the results were described almost properly. In this meaning, the manuscript is sound and suitable for the indexing. However, as I mentioned below, this manuscript has still some points to be clearly explained.

1. Comparison of the genome sequences of human bocaviruses between from Vietnam and the others should be much more discussed.
2. To date, all of the HBoV genotypes contain the episomal structure. Then, it would be better to analyze it in the paper.
3. The genome of HBoV is organized in three ORFs: ORF1 encoding NS1 protein; ORF2 encoding NP1 protein; ORF3 encoding VP1 and VP2 proteins. So it was suggested that the Phylogenetic trees of nucleotide and amino acid sequences of the HBoV genes should be constructed.

I have read this submission. I believe that I have an appropriate level of expertise to confirm that it is of an acceptable scientific standard, however I have significant reservations, as outlined above.

# Response

**Published:** 2017-01-03

**Authors:** Thanh Tran Tan

**Affiliations:** Oxford University Clinical Research Unit, Vietnam

1. Comparison of the genome sequences of human bocaviruses between from Vietnam and the others should be much more discussed. 
**Response**:
  We have now discussed this in the discussion section. The second sentence of the discussion reads “Phylogenetically, the four HBoVs from Vietnam were closely related to other HBoV strains sampled from various countries worldwide, reflecting a wide distribution of these HBoV lineages at global scales”.
2. To date, all of the HBoV genotypes contain the episomal structure. Then, it would be better to analyze it in the paper. 
**Response**:
  We thank the referee for this comment. Please forgive our ignorance but we understood that episomal structure is formed by repeated sequences at the 5’ and 3’ ends, which unfortunately were not fully sequenced. Therefore the analysis could not be done reliably.
3. The genome of HBoV is organized in three ORFs: ORF1 encoding NS1 protein; ORF2 encoding NP1 protein; ORF3 encoding VP1 and VP2 proteins. So it was suggested that the Phylogenetic trees of nucleotide and amino acid sequences of the HBoV genes should be constructed. 
**Response**:
  We have reconstructed additional phylogenetic trees according to the suggestion of Dr Xiu-ling Ji. We have therefore added those additional phylogenetic to this revised version, and added a sentence to elaborate it in the result section; “Similar results were obtained when the analyses were done for 3 individual open reading frames, ORF1, ORF2 and ORF3 (Figure 1)”.

# Peer review: Referee response for version 1

**DOI:** 10.21956/wellcomeopenres.10819.r17686

**Published:** 2016-12-02

**Authors:** Verena Schildgen, Oliver Schildgen

**Affiliations:** 1 Department of Pathology, gGmbH Clinic of Cologne, Hospital of University of Witten/Herdecke, Cologne, Germany

Although the article adds novel information on HBoV epidemiology in Vietnam and presents the sequences of current strains in this geographic region, it has numerous technical shortcomings. The methodology used is not sufficiently described. E.g., the authors state that coxsackieviruses were detected by whole genome sequencing. This technique, however, would detect genomic host DNA, coxsackie is an RNA virus. Moreover, primer sequences and PCR protocols are missing, and alignments are not shown. The reference list is extremely short, and the overall description of methods, results and discussion is weak.

We have read this submission. We believe that we have an appropriate level of expertise to confirm that it is of an acceptable scientific standard, however we have significant reservations, as outlined above.

# Response

**Published:** 2017-01-03

**Authors:** Thanh Tran Tan

**Affiliations:** Oxford University Clinical Research Unit, Vietnam

**Response**:

 We thank Dr Verena Schildgen and Dr Oliver Schildgen for their constructive comments **. **Please allow us to clarify that the method used was developed in our laboratory for amplification and sequencing viral sequences, and it has been published (citation #11). Although host DNA can also be simultaneously sequenced, investigation of its presence in the obtained reads is beyond the scope of the present Research Note.

 Given the method used was detailed in our previous publication (including primer sequences), we have chosen to briefly present it in this revised version as per the reviewers’ comment.  Likewise, we have updated our reference list (from 11 to 23 references), and added more text to the discussion section. Please also refer to our responses to the other reviewers regarding the updated result section, while we hope the reviewers appreciate that the format of a Research Note is concise.

# Peer review: Referee response for version 1

**DOI:** 10.21956/wellcomeopenres.10819.r17741

**Published:** 2016-11-21

**Authors:** Bas B. Oude Munnink

**Affiliations:** 1 Viroscience, Erasmus MC, Rotterdam, Netherlands

This paper describes the first complete genome sequences of human bocaviruses in Vietnam. Reporting complete genome sequences from potential local viral pathogens is vital to develop accurate diagnostic methods and to perform additional studies. However, depending on the scope of this journal, this paper would also be suitable for publication in the journal Genome Announcements.

 The paper is very compact and carefully written, however I feel that some essential information is missing:

- The authors show that they have detected 4 bocaviruses in enterovirus positive samples, as identified by RT-PCR, which demonstrates the strength of agnostic deep sequencing. However, could it be that the symptoms observed in these patients was caused by these enteroviruses and not by the bocaviruses detected in these samples? And which specific enteroviruses (or other viral pathogens) were detected in these bocavirus positive samples? 
- Three viruses were found in throat swabs while one virus was found in rectal swabs. Which virus was found in which sample? E.g. was the genome coverage in the rectal swab lower and does this perhaps also explain the different species detected — was species 2 found in rectal swabs and species 1 in throat swabs?

And some minor points:

- In the abstract the authors mention that “The sequences may aid future study aiming at understanding the evolution of the pathogen”. However, in the introduction and conclusion they mention that the clinical significance of bocavirus infection remains unknown. I suggest to change the word pathogen by virus. 
- The phylogenetic tree is difficult to read in the current resolution

I have read this submission. I believe that I have an appropriate level of expertise to confirm that it is of an acceptable scientific standard, however I have significant reservations, as outlined above.

# Response

**Published:** 2017-01-03

**Authors:** Thanh Tran Tan

**Affiliations:** Oxford University Clinical Research Unit, Vietnam

1. The authors show that they have detected 4 bocaviruses in enterovirus positive samples, as identified by RT-PCR, which demonstrates the strength of agnostic deep sequencing. However, could it be that the symptoms observed in these patients was caused by these enteroviruses and not by the bocaviruses detected in these samples? And which specific enteroviruses (or other viral pathogens) were detected in these bocavirus positive samples? 
**Response:**
  We agree with the referee that the observed signs/symptoms might have been caused by HFMD causing enteroviruses, and have discussed this in the discussion section added to this revised version.
  We have provided the information about the specific enteroviruses detected in the four HFMD in the text and Table 1 added to this revised version. The second sentence from the last of the result section now reads “All the four HFMD patients (including 3 CV-A6 and 1 CV-A12, Table 1) in whom HBoV was detected had mild HFMD, and were enrolled in November 2013 – March 2014.”
2. Three viruses were found in throat swabs while one virus was found in rectal swabs. Which virus was found in which sample? E.g. was the genome coverage in the rectal swab lower and does this perhaps also explain the different species detected — was species 2 found in rectal swabs and species 1 in throat swabs? 
**Response:**
  We found HBoV-1 in 3 throat swabs and HBoV-2 in 1 rectal swab. There was no correlation between genome coverage and sample types (i.e. rectal/throat swab), although the sample size was small. We have presented those data in the original manuscript and have now modified the text slightly to further elucidate the referee’s comment and provided the details in Table 1.
  “Evidence of bocavirus sequences were found in four swabs (including 3 throat- and 1 rectal swabs). A reference-based mapping approach using Geneious software (Biomatters) was then employed to recover the HBoV genomes from the corresponding dataset. Subsequently, 2 CDS (1 from a throat swab with 4925 bp in length and the other from a rectal swab with 4898bp; i.e. over 90% of genome coverage) were successfully assembled with a mean coverage of 1,922 and 3,745, respectively (Table 1). In the other datasets from the remaining two swabs only partial genomic sequences of HBoV, each with 2870bp in length and a mean coverage of 15.4 and 448.7, were recovered.
  Subsequent sequence alignment and phylogenetic analysis using MUSCLE and Neighbor-joining available in Geneious (Biomatters), respectively (Figure 1) revealed that all 3 Vietnamese HBoV recovered from the throat swabs belonged to HBoV-1 and had >98% of sequence similarity at nucleotide level with other HBoV-1. The other recovered from rectal swab belonged to HBoV-2 and had a close relatedness with a Thai strain CU54TH (GU048663) with a sequence similarity of 97.3% (Figure 1).”
  Additionally, we have also discussed the detection of HBoV-1 and HBoV-2 in throat- and rectal swab, respectively in the discussion section.
3. In the abstract the authors mention that “The sequences may aid future study aiming at understanding the evolution of the pathogen”. However, in the introduction and conclusion they mention that the clinical significance of bocavirus infection remains unknown. I suggest to change the word pathogen by virus. 
**Response:**
  We have replaced the word “pathogen” by “virus” as recommended.
4. The phylogenetic tree is difficult to read in the current resolution
**Response:**
  We have improved the resolution of the phylogenetic trees.
